# The Kimberley Assessment of Depression of Older Indigenous Australians: Prevalence of Depressive Disorders, Risk Factors and Validation of the KICA-dep Scale

**DOI:** 10.1371/journal.pone.0094983

**Published:** 2014-04-16

**Authors:** Osvaldo P. Almeida, Leon Flicker, Stephen Fenner, Kate Smith, Zoe Hyde, David Atkinson, Linda Skeaf, Roslyn Malay, Dina LoGiudice

**Affiliations:** 1 School of Psychiatry & Clinical Neurosciences, University of Western Australia, Perth, Australia; 2 WA Centre for Health & Ageing, Centre for Medical Research, Perth, Australia; 3 Department of Psychiatry, Royal Perth Hospital, Perth, Australia; 4 Department of Geriatric Medicine, Royal Perth Hospital, Perth, Australia; 5 School of Medicine and Pharmacology, University of Western Australia, Perth, Australia; 6 Rural Clinical School, University of Western Australia, Perth, Australia; 7 Kimberley Aboriginal Medical Services, Broome, Australia; 8 Aged Care, Melbourne Health, Melbourne, Australia; Institute of Neuroepidemiology and Tropical Neurology, France

## Abstract

**Objective:**

This study aimed to develop a culturally acceptable and valid scale to assess depressive symptoms in older Indigenous Australians, to determine the prevalence of depressive disorders in the older Kimberley community, and to investigate the sociodemographic, lifestyle and clinical factors associated with depression in this population.

**Methods:**

Cross-sectional survey of adults aged 45 years or over from six remote Indigenous communities in the Kimberley and 30% of those living in Derby, Western Australia. The 11 linguistic and culturally sensitive items of the Kimberley Indigenous Cognitive Assessment of Depression (KICA-dep) scale were derived from the signs and symptoms required to establish the diagnosis of a depressive episode according to the DSM-IV-TR and ICD-10 criteria, and their frequency was rated on a 4-point scale ranging from ‘never’ to ‘all the time’ (range of scores: 0 to 33). The diagnosis of depressive disorder was established after a face-to-face assessment with a consultant psychiatrist. Other measures included sociodemographic and lifestyle factors, and clinical history.

**Results:**

The study included 250 participants aged 46 to 89 years (mean±SD = 60.9±10.7), of whom 143 (57.2%) were women. The internal reliability of the KICA-dep was 0.88 and the cut-point 7/8 (non-case/case) was associated with 78% sensitivity and 82% specificity for the diagnosis of a depressive disorder. The point-prevalence of a depressive disorder in this population was 7.7%; 4.0% for men and 10.4% for women. Heart problems were associated with increased odds of depression (odds ratio = 3.3, 95% confidence interval = 1.2,8.8).

**Conclusions:**

The KICA-dep has robust psychometric properties and can be used with confidence as a screening tool for depression among older Indigenous Australians. Depressive disorders are common in this population, possibly because of increased stressors and health morbidities.

## Introduction

Depression is a leading cause of years lived with disability [1], affecting about 5% of the population at any point in time [2], including older adults [3]. Some investigators have suggested that depression is more frequent among people living in rural than urban areas [4], [5], although the consistency of these associations has been questioned [6] and may vary according to the cultural background of participants. There are concerns, for example, that depression may be particularly common among Indigenous Australians living in remote areas [7], but surveys of these populations have been opportunistic and have used strategies of uncertain validity to ascertain the presence of depression [8], [9].

An early attempt to validate a modified version of the Patient Health Questionnaire (PHQ-9) for use in this population recruited 34 Indigenous people with ischaemic heart disease in contact with a specific health service of the Northern Territory of Australia [10]. They reported that the PHQ-9 cut-point 8/9 (non-case/case) was associated with 70% sensitivity, 78% specificity, 58% positivity predictive value and 86% negative predictive value for the diagnosis of either minor or major depression. They also found that clinically significant depressive symptoms affected nine of the 32 (28%) participants who completed an additional clinical assessment [10]. Limitations of that study included its small sample size, unrepresentative nature of the sample, and uncertain validity of the GP-derived diagnosis of depression. More recently, an adapted version of the PHQ-9 was developed for use among Indigenous men living in central Australia [11], but data regarding its psychometric characteristics and validity were not available.

Two critical issues remain to be resolved in order to establish the true prevalence of depressive disorders among Indigenous Australians: (1) development of a screening tool with robust psychometric properties, (2) evaluation of a representative sample of older Indigenous people living in rural and remote areas of Australia. The Kimberley Indigenous Cognitive Assessment of Depression (KICA-dep) was a sub-project of the ‘Kimberley Healthy Ageing Project’ designed to develop a culturally acceptable and valid scale to assess depressive symptoms among all older Indigenous people living in six Kimberley remote communities (plus a random sample of Indigenous people living in Derby), determine the prevalence of depressive disorders among older Indigenous people living in the Kimberley, investigate the sociodemographic, lifestyle and clinical factors associated with depression in this population.

## Methods

### Study design and setting

Cross-sectional study of older Indigenous people living in rural and remote areas of Western Australia and in Derby.

### Participants

Participants were Indigenous Australians aged 45 years or over living in the communities of Mowanjum, Warmun, Ardyaloon, Junjuwa, Looma and Wirrimanu. We also recruited a 33% pseudo-random sample of those living in Derby (every third potentially eligible person on the list, including people living in residential care facilities). We excluded from this study volunteers who showed evidence of cognitive impairment, as established by a KICA-cog score <32 [12]. The identification of potentially eligible participants was facilitated by relevant community leaders and assistants.

We obtained approval to complete this study from the appropriate community councils, the Kimberley Aboriginal Medical Services Council, Kimberley Aged and Community Services and from the Human Research Ethics Committee of the University of Western Australia and Western Australia Aboriginal Health Ethics Committee. All participants provided written informed consent.

### The Kimberley Indigenous Cognitive Assessment of Depression (KICA-dep) scale

Items were derived from the signs and symptoms required to establish the diagnosis of a depressive episode according to the DSM-IV-TR and ICD-10 criteria, and followed the same format of the Patient Health Questionnaire [13]. Each item was rated on a frequency scale that ranged from ‘never’ to ‘sometimes’, ‘a lot’ and ‘all the time’, with the respective assigned scores of zero, one, two and three. The wording of each item was revised for linguistic and culture relevance by the research team and community assistants, and additional items were included if existing statements were considered insufficient to cover a relevant domain. For example, the question about suicide ideation was presented as two separate questions in order to facilitate rating: “Have you had thoughts that you would be better off dead?” and “Have you thought of hurting yourself?”. A copy of the KICA-dep scale appears in table 1. For the purposes of this study, the research assistant read aloud each of the 11 items of the KICA-dep to participants, who were then asked to rate the frequency with which they had experienced each item during the preceding week.

**Table 1:** Prevalence of KICA-dep items among Indigenous Australians with and without ICD-10 or DSM-IV-TR diagnosis of depression.

KICA-Dep Items:		Clinical Depression according to ICD-10 and DSM-IV criteria*	
*In the last week have you:*	Response	No	Yes
		n = 126	n = 18
		N (%)	N (%)
*Felt down, sad, no good?*	Never	71 (56.3)	2 (11.1)
	Sometimes	48 (38.1)	11 (61.1)
	A lot	2 (1.6)	1 (5.6)
	All the time	5 (4.0)	4 (22.2)
*Felt like doing things that you usually like doing? (things that make you happy)*	Never	74 (58.7)	2 (11.1)
	Sometimes	47 (37.3)	12 (66.7)
	A lot	3 (2.4)	3 (16.7)
	All the time	2 (1.6)	1 (5.6)
*Had trouble getting to sleep, staying asleep, or sleeping too much?*	Never	92 (73.0)	3 (16.7)
	Sometimes	26 (20.6)	8 (44.4)
	A lot	2 (1.6)	2 (11.1)
	All the time	6 (4.8)	5 (27.8)
*Felt more tired or slack, like you've had no energy?*	Never	49 (38.9)	4 (22.2)
	Sometimes	67 (53.2)	9 (50.0)
	A lot	4 (3.2)	2 (11.1)
	All the time	6 (4.8)	3 (16.7)
*Been eating too much or eating only a little bit?*	Never	103 (81.7)	11 (61.1)
	Sometimes	18 (14.3)	5 (27.8)
	A lot	1 (0.8)	1 (5.6)
	All the time	4 (3.2)	1 (5.6)
*Felt bad about yourself, or felt shamed that you have let yourself or family down*	Never	88 (69.8)	6 (33.3)
	Sometimes	32 (25.4)	5 (27.8)
	A lot	2 (1.6)	1 (5.6)
	All the time	4 (3.2)	6 (33.3)
*Had trouble paying attention, or concentrating on things?*	Never	96 (76.2)	5 (27.8)
	Sometimes	24 (19.0)	9 (50.0)
	A lot	2 (1.6)	1 (5.6)
	All the time	4 (3.2)	3 (16.7)
*Been told that you are speaking or moving too slowly or fast?*	Never	95 (75.4)	8 (44.4)
	Sometimes	23 (18.2)	6 (33.3)
	A lot	3 (2.4)	1 (5.6)
	All the time	5 (4.0)	3 (16.7)
*Had thoughts that you would be better off dead?*	Never	111 (88.1)	5 (29.4)
	Sometimes	13 (10.3)	8 (47.1)
	A lot	0	2 (11.8)
	All the time	2 (1.6)	2 (11.8)
*Thought of hurting yourself?*	Never	116 (92.1)	6 (33.3)
	Sometimes	13 (10.3)	7 (38.9)
	A lot	0	2 (11.1)
	All the time	2 (1.6)	3 (16.7)
*Felt wild (angry)?*	Never	68 (54.0)	3 (16.7)
	Sometimes	53 (42.1)	11 (61.1)
	A lot	2 (1.6)	1 (5.6)
	All the time	3 (2.4)	3 (16.7)

*ICD-10 codes F32 or F43, DSM-IV-TR codes 296, 309 or 311.

All participants with a KICA-dep score of 9 or more were invited to undergo a clinical review with a consultant psychiatrist (SF) to ascertain the presence of a psychiatric syndrome such as a major depressive episode. Participants with PHQ-9<9 who acknowledged suicidal ideation were also invited. Every third participant with a KICA-dep <9 received a similar invitation - this enabled us to generate a study sample with broad distribution of scores to investigate the psychometric properties of the scale.

### Establishing the point-prevalence of depressive disorders

One-hundred and forty-four participants underwent a comprehensive clinical review with a consultant psychiatrist, who documented relevant signs and symptoms and ascribed ICD-10 and DSM-IV-TR diagnoses, which were later reviewed by an independent physician (LF). Discrepancies of diagnoses were discussed at a meeting of the investigators until consensus was reached. The diagnosis of depressive disorders included codes F32 and F43 for ICD-10, and codes 296, 309 and 311 for DSM-IV-TR. Another 91 participants who were not invited to the face-to-face assessment with the psychiatrist were later included in the prevalence study sample as ‘non-cases’ because they had a KICA-dep score <9 (high negative predictive value - see below). We excluded from these analyses 15 people KICA-dep ≥9 who declined further assessment with the psychiatrist (average positive predictive value - see below). For the purposes of this study, the term ‘point-prevalence’ is used to indicate that a depressive disorder was present at the time of assessment. Prevalence data were summarised in percentages (i.e., the number of people with depression out of 100 people at risk).

### Other study measures

We used a semi-structured interview to ascertain the age of participants in years (date of the interview minus the date of birth, divided by 365.25), their gender, preferred language, education [*Did you go to school?* (*yes/no*)], smoking [*Do you smoke? (yes/no) Did you smoke when you were younger? (yes/no)*] and alcohol use history [*Do you drink grog? (yes/no); How many times a week? (only sometimes, not every week, 1–3 days a week, 4–6 days a week, everyday); How much do you usually drink? (just a few drinks: 1–3 drinks, a lot: 4–6 drinks, big mob: until drunk)*]. We considered that participants who reported consuming four or more daily drinks at least 4–6 times per week were consuming alcohol at hazardous or harmful levels. We measured participants' height (to 0.1 cm) and weight (to 0.1 Kg), which we used to calculate their body mass index (BMI, Kg/m^2^). They were then classified as underweight (BMI<18.5), normal (18.5≤BMI<25), overweight (25≤BMI≤30) or obese (BMI≥30). Participants also reported whether they had kidney or heart problems (yes/no/don't know to both), and whether they had ‘*ever been hit on the head and knocked out*’ (yes/no/don't know). We considered that persistent pain was present if they answered ‘*all the time*’ to the question ‘*Do you ever have any pain*?’ In addition, participants reported whether they ‘*had sugar sickness (diabetes)*’ or ‘*high blood pressure*’ (yes/no/don't know). We considered diabetes to be present if they answered ‘yes’ to the question about sugar sickness or if they had a glycated haemoglobin (HbA1c) blood test >7% at the time of assessment. Similarly, we classified people as hypertensive if they acknowledged high blood pressure or if they had a measured systolic blood pressure ≥140 mmHg or a diastolic blood pressure ≥90 mmHg at the time of the assessment.

### Statistical analyses

Data were managed and analyzed with the statistical package Stata release 13.1 (StataCorp, College Station, TX). We used descriptive statistics (mean, standard deviation of the mean [SD], proportions) to summarize the data, and Pearson's chi-square statistic (χ^2^) to compare the distribution of various measured factors according to participants' depression status. We used Cronbach's alpha to evaluate the internal consistency (reliability) of the KICA-dep, and two by two tables to estimate sensitivity, specificity, positive predictive value (PPV) and negative predictive value (NPV) associated with different cut-points of the scale. We generated a receiver operating characteristic (ROC) curve using the ‘roctab’ command of Stata, which allowed us to estimate the area under the ROC curve. We estimated the point-prevalence of depression by dividing the number of people with a depressive disorder by the total number of people in the sample, and calculated the 95% confidence interval (95%CI) of the prevalence estimate using generalized linear modeling (glm). The odds ratios (OR) of depression and respective 95%CI associated with relevant exposures were calculated using logistic regression. Alpha was set at 5% and all probability tests reported were two-tailed.

## Results

Three hundred and ninety-four people were invited to take part in the study. Of these, 37 were away, 4 could not be located, 46 refused, 15 had relocated, and in 3 cases the reason for non-participation was uncertain, leaving 289 people. Of the 289 people who consented and met the inclusion criteria for participation, 22 were subsequently excluded because they showed evidence of cognitive impairment (KICA-cog <32) and another 17 did not complete the assessment with the KICA-dep, leaving a study sample of 250 participants. Their mean age was 60.9 years (SD = 10.7; range 46 to 89) and 143 (57.2%) were women. Table 2 shows the basic sociodemographic, lifestyle and clinical characteristics of participants.

**Table 2:** Sociodemographic, lifestyle and clinical characteristics of older Indigenous Australians.

		N = 250; N (%)
Aged 45 to 59 years		147 (58.8)
Female gender		143 (57.2)
Education: any schooling		205 (82.0)
Hazardous or harmful alcohol use ^1^		79 (31.6)
Smoking history ^1^	Never	86 (34.4)
	Current	73 (29.2)
	Past	90 (36.0)
Body mass index group ^38^	Normal	61 (24.4)
	Underweight	12 (4.8)
	Overweight	69 (27.6)
	Obese	70 (28.0)
Past head injury ^7^		73 (29.2)
Hypertension ^19^		160 (64.0)
Diabetes ^13^		167 (66.8)
Heart problems ^11^		64 (25.6)
Kidney problems ^18^		62 (24.8)
Persistent pain ^3^		52 (20.8)

n Number of participants for whom data were missing.

### KICA-dep: internal consistency and validity of the scale

**Figure 1:**
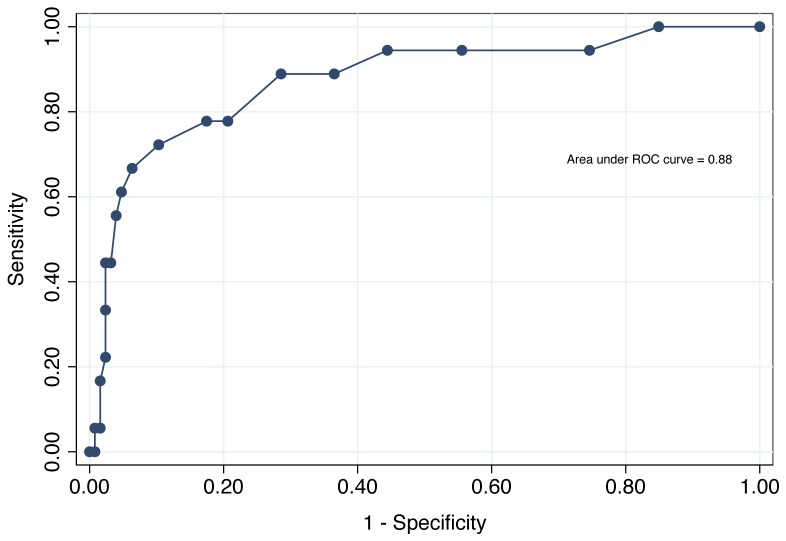
Receiver operating characteristic (ROC) curve of KICA-Dep scores for the diagnosis of any depressive disorder according to ICD-10 Criteria for Research. A KICA-Dep cut-point of 7/8 (non-case/case) was associated with sensitivity of 78%, specificity of 82%, positive predictive value of 39% and negative predictive value of 96%. Sensitivity and specificity values for other cut-points were 89% and 63% for 4/5, 89% and 71% for 5/6, 78% and 80% for 6/7, 72% and 90% for 8/9, and 67% and 94% for 9/10.

The intraclass correlation of KICA-dep items was 0.88, showing that the scale had excellent internal consistency. One hundred and forty-four of the 250 participants completed an additional face-to-face assessment with a consultant psychiatrist who was blind to the results of the KICA-dep. Invitations for this assessment were issued to all participants with KICA-dep total score ≥9 and to every third participant with KICA-dep <9. This clinical examination, which took place within no more than four weeks of the assessment with the KICA-dep, led to a clinical diagnosis according to ICD-10 and DSM-IV-TR criteria. Table 1 shows the frequency distribution of individual KICA-dep items for participants with and without a depressive disorder. Figure 1 shows the receiver operating characteristic (ROC) curve of KICA-dep scores according to a ICD-10 or DSM-IV-TR diagnosis of depression (we did not complete further analyses with specific diagnostic groups because of small numbers in the cells). The area under the curve was 0.88. For the cut-point 7/8 (non-case/case), the sensitivity was 78%, specificity 82%, PPV 39% and NPV 96%, whereas for the cut-point 8/9, sensitivity was 72%, specificity 90%, PPV 50% and NPV 96%.

### Point prevalence of depression among older Indigenous Australians

Fifty-six (22.4%) of the 250 participants who completed the KICA-dep had a total score ≥8 and 41 (16.4%) a score ≥9. Given the high NPV and average PPV of the KICA-dep cut-point 8/9 for the diagnosis of a depressive disorder (i.e., very few false negatives but several false positive cases), we ascribed a diagnosis of ‘no depression’ for participants who had completed the KICA-dep and had a total score <9 (all cases not referred for assessment with the psychiatrist). The 15 participants with KICA-dep ≥9 who declined a psychiatric review were excluded from further analysis due to uncertainty about their clinical status. Of the remaining 235 participants, 18 fulfilled criteria for a depressive disorder, resulting in a point-prevalence of 7.7% (95%CI = 4.3%, 11.1%). The prevalence of a current depressive disorder was 4.0% (95%CI = 0.1%, 7.8%) for men and 10.4% (95%CI = 5.2%, 15.6%) for women. According to ICD-10 criteria, of the 18 people with a depressive disorder, 14 had a depressive episode and 4 an adjustment disorder, while according to DSM0IV-TR criteria 10 had major depression, 4 adjustment disorder and 4 depressive disorder not otherwise specified.

### Factors associated with depression in older Indigenous Australians

Table 3 summarises the sociodemographic, lifestyle and clinical characteristics of Indigenous Australians with and without depression. Heart problems were more prevalent among participants with than without depression (OR = 3.31, 95%CI = 1.24,8.81). There was also a non-significant excess of women, and people with diabetes and kidney diseases amongst those with compared with those without depression (table 3).

**Table 3:** Sociodemographic and clinical characteristics of Indigenous Australians older than 45 years with and without a current depressive disorder.

		No depression	Depression	Statistic	p-value
		N = 217	N = 18	X^2^	
		N (%)	N (%)		
Aged 45 to 59 years		123 (56.7)	11 (61.1)	0.13 (1)	0.715
Female gender		120 (55.3)	14 (77.8)	3.43 (1)	0.064
Education: any schooling		173 (79.7)	17 (94.4)	2.33 (1)	0.127
Hazardous or harmful alcohol use		64 (29.6) ^1^	5 (27.8)	0.03 (1)	0.869
Smoking history	Never	78 (36.1)	7 (38.9)	0.45 (2)	0.798
	Past	64 (29.6)	4 (22.2)		
	Current	74 (34.3) ^1^	7 (38.9)		
Body mass index group	Normal	56 (30.4)	3 (18.7)	3.21 (3)	0.361
	Underweight	10 (5.4)	0		
	Overweight	57 (31.0)	8 (50.0)		
	Obese	61 (33.1) ^33^	5 (31.2) ^2^		
Past head injury		60 (28.3) ^5^	6 (37.5) ^2^	0.61 (1)	0.434
Hypertension		139 (68.4) ^14^	13 (76.5) ^1^	0.47 (1)	0.493
Diabetes		140 (68.3) ^12^	16 (88.9)	3.34 (1)	0.068
Heart problems		48 (23.2) ^10^	9 (50.0)	6.29 (1)	0.012
Kidney problems		49 (24.4) ^16^	8 (44.4)	3.46 (1)	0.063
Persistent pain		40 (18.7) ^3^	5 (27.8) ^1^	0.88 (1)	0.349

df = degrees of freedom.

n number of participants in the group for whom data were missing.

## Discussion

The KICA-dep, a scale akin to the PHQ-9, has robust psychometric properties and can be used effectively to screen for the presence of depression among older Indigenous Australians. The high NPV of the KICA-dep associated with scores lower than 8 indicates that the scale is excellent at screening out people without depression, although the modest PPV associated with scores of 8 or more highlight the need for a detailed clinical assessment before the presence of clinically significant depressive symptoms can be established with confidence. We also found that the prevalence of a depressive disorder among Indigenous Australians older than 45 years is 7.7% (6.0% for major depression), and that diseases of the heart increase the odds of depression in this population.

Before discussing the implications of these results, we should consider the merits and limitations of the study. We identified and approached all Indigenous people aged 45 years or over living in 6 remote communities of Western Australia, in addition to about 30% of those living in the town of Derby. Seventy-three percent of those approached agreed to participate, giving us confidence that our results can be generalized to these communities. In addition, the items of the scale we used to screen for the presence of depression were derived from existing diagnostic guidelines and, although they were modeled on the PHQ-9, they were worded and presented in a culturally sensitive manner. The KICA-dep showed excellent internal consistency and good face-validity as a screening instrument for depressive disorders according to DSM-IV-TR and ICD-10 criteria. We concede, however, that the assessment of mental state carried out by our experienced consultant psychiatrist did not rely on the use of a structured clinical interview. The study also has the merit of having assessed relevant sociodemographic, lifestyle and clinical factors using a systematic and semi-structured approach that optimised self-reported information with direct measures, such as HbA1c and blood pressure measurements. However, we acknowledge that the cross-sectional design of the study provides only a snapshot of the mental state of Indigenous Australians and that the time-lag between assessment with the KICA-dep and review with the consultant psychiatrist ranged from one day to 4 weeks. As changes in mood could have occurred between these two assessments, it is conceivable that some of the observed characteristics of the KICA-dep could have been affected by this time difference (such as its sensitivity and PPV). A sensitivity analyses could have provided useful information in this regard, but the relatively small number of participants with depression in this sample hindered our ability to do so. We also recognize that we cannot be certain about the validity of the self-reported use of alcohol, smoking, past head injury, and heart and kidney problems, although other studies have reported similar lifestyle practices in other Indigenous populations [9] - whether consistency is a good indicator of accuracy in this instance remains to be determined. Finally, one might argue that the point-prevalence of depressive disorders that we observed in our sample is not an estimate but a real measure, as we assessed nearly all eligible participants of the targeted communities. Notwithstanding the value of such an argument, we chose to adopt a conservative approach to our analyses because of refusals and the inclusion of a sample of Indigenous living in Derby. Hence, our prevalence estimates are accompanied by confidence limits that highlight the uncertainty surrounding our calculations.

This study set out to develop a culturally sensitive scale for the screening of depression among older Indigenous Australians living in remote and rural areas. Our results show that the KICA-dep has robust psychometric properties and that the use of the cut-point 7/8 is excellent at discriminating people without depression from those who require further assessment. An equally low PPV for an adapted version of the PHQ-9 was reported by others in a small sample of 34 Northern Territory Indigenous people with ischaemic heart disease [10]. Consequently, the KICA-dep should not be used to establish ‘caseness’, although it can be used with confidence for ‘non-caseness’. The KICA-dep is brief, easy to use and was well accepted by participants.

Another aim of this study was to determine how common depressive disorders were in this population of older Indigenous people. The observed point-prevalence of 7.7% is very similar to the frequency of clinically significant depression found in the Australian population aged 60 years or over [14], although the proportion of people with major depression in our sample was at least twice as high as in the non-Indigenous community. A recent survey of 1939 Indigenous Australians aged 45 years or over from New South Wales and the Torres Strait Islands found that 23% of the Indigenous population reported past history of depression compared with 13% among non-Indigenous people (n = 259984) [9]. Whether the higher prevalence of depression among Indigenous Australians occurs both in rural and urban settings remains to be established.

It is surprising that most cases identified by the consultant psychiatrist were classified as major depression or depressive episodes. There are two possible ways of interpreting this finding: (1) disorders such as dysthymia, depression due to general medical conditions or mood disorder not otherwise specified are uncommon among Indigenous Australians, (2) the unstructured clinical assessment with the psychiatrist had poor sensitivity for the diagnosis of non-major depressive disorders. Given the higher than expected prevalence of major depressive episodes and the relatively large proportion of participants with KICA-dep scores ≥8, we are unable to dismiss the latter possibility. Should that be the case, then the prevalence of depressive disorders in this community may be at least twice as high as in the general Australian population, and the PPV of the KICA-dep might be more robust than reported. Future studies should use a structured clinical interview for the assessment of mental state to ascertain with greater confidence the prevalence of non-major depressive disorders among Indigenous Australians. This recommendation is not a mere academic exercise: non-major depressive disorders are associated with substantial disability and increased costs [3], [15], [16], thus having confidence about their true prevalence in the community is critical for the development of sound health planning and policy.

We found that the prevalence of other medical morbidities, such as diabetes and heart problems, was very high in this population compared with general Australian community [17]. Depression was more frequent among people with than without heart problems, and there was also a non-significant excess among people with diabetes and kidney diseases. This pattern of increased health burden suggests an earlier onset of frailty among Indigenous Australians [18], and is consistent with previous findings of higher prevalence and earlier onset of dementia in these communities [19]. As physiological and environmental stresses increase the risk of depression [20], a high prevalence of depression among older Indigenous people could be expected. In addition, these results suggest that health promotion strategies that are effective at decreasing the burden associated with diabetes, kidney and heart diseases may also contribute to lower the prevalence of depression in these communities. The reverse could also be true: the successful identification and management of depression could decrease the incidence of other health morbidities, such as cardiovascular events [21], [22], in this at risk population.

In conclusion, the KICA-dep has robust psychometric properties and can be used with confidence as a depression screening tool for Indigenous Australians. We found that depressive disorders are common and that major depression is at least twice as frequent in this population as in the non-Indigenous community, possibly as a result of increased stressors and health morbidities, such cardiac conditions. The contribution of non-major depressive disorders to the disability of this population requires further investigation, as does the response of people with depression to standard antidepressant treatment.
